# Investigating the impact of *IKZF1* SNPs rs4132601 and rs11978267 on acute lymphoblastic leukemia: a comprehensive meta-analysis

**DOI:** 10.1186/s43046-025-00274-2

**Published:** 2025-04-11

**Authors:** Sheena Mariam Thomas, Jethendra Kumar Muruganantham, Praveen Kumar Chandra Sekar, B. K. Iyshwarya, Ramakrishnan Veerabathiran

**Affiliations:** Human Cytogenetics and Genomics Laboratory, Faculty of Allied Health Sciences, Chettinad Hospital and Research Institute, Chettinad Academy of Research and Education, Kelambakkam, Tamil Nadu 603103 India

**Keywords:** Leukemias, Acute lymphoblastic leukemia, *IKZF1* Gene, Single nucleotide polymorphisms, Heterogeneity, Genetic association

## Abstract

**Objective:**

This meta-analysis investigates the association between acute lymphoblastic leukemia (ALL) susceptibility and *IKZF1* gene SNPs.

**Methods:**

Utilizing EMBASE, PubMed, and other databases, the study evaluated methodological quality through the Newcastle–Ottawa Scale (NOS) scoring and Hardy–Weinberg Equilibrium (HWE) value. The present meta-analysis used Preferred Reporting Items for Systematic Reviews and Meta-analysis (PRISMA) guidelines. Review Manager 5.4 software was employed for data analysis, emphasizing genetic variants' significance (*p* < 0.05). Visualizations were achieved using funnel and Circos plots.

**Results:**

A significant association was found between rs4132601 and ALL across genetic models, contrasting with the non-significant correlation for rs11978267. The findings underscore the complex interplay of genetic factors in ALL susceptibility, particularly related to *IKZF1* SNPs. Ethnicity emphasizes the importance of diverse population considerations.

**Conclusion:**

This meta-analysis highlights the significance of rs4132601 in ALL's genetic foundation, suggesting potential advancements in diagnostics. The lack of correlation for rs11978267 highlights the complexity of its genetic association. Future studies should prioritize larger, diverse samples for a comprehensive understanding and improved strategies for ALL diagnoses and treatments.

## Introduction

Leukemias are a diverse group of hematologic disorders primarily affecting white blood cells, with varying clinical presentations, prognoses, and responses to treatment [1]. These disorders are characterized by an abnormal population of cells that disrupt the normal production of cellular components in the hematological system [2]. Leukemias originate from hematopoietic stem cells (HSCs) and their precursors in the bone marrow (BM), promoting the proliferation and infiltration of leukemic cells [3]. Hematological cancers are commonly classified based on cellular involvement in the bone marrow, the degree of cell maturation, and lineage. Myeloid lineage neoplasms include granulocytes (neutrophils, eosinophils, and basophils), monocytes, erythrocytes, platelets (derived from megakaryocytes), and mast cells. In contrast, T, B, and NK cells are associated with the lymphoid lineage [4].

ALL is a B or T-cell lineage malignancy arising from a single hematopoietic precursor. While some cases are linked to predisposing factors such as inherited genetic susceptibility and environmental exposures, the majority of individuals with ALL do not have any pre-existing conditions. ALL is characterized by chromosomal abnormalities and genetic alterations that affect precursor cells responsible for lymphoid development and proliferation [5]. Among new cancer diagnoses in the United States, ALL represents a severe hematologic malignancy with an estimated incidence of 0.3%. It is the most common cancer in children under 15, accounting for approximately 20% of all pediatric malignancies [6].

The disease can begin with a chromosomal translocation, but symptoms usually appear when leukemic progenitors in the bone marrow collect more genetic abnormalities and deletions that drive disease development. Deletions, mutations, or large chromosomal rearrangements involving the B-cell transcription factor *IKZF1* are critical prognostic markers of poor outcomes in ALL. *IKZF1* serves as a vital regulator of B-cell development and a tumor suppressor in leukemia, with increasing evidence suggesting that its loss affects signaling pathways that influence therapeutic responses [7]. The IKAROS protein, encoded by the *IKZF1* gene located on chromosome 7, interacts with deoxynucleotidyl transferase to regulate protein transcription, particularly targeting the dead centromeric chromosomal zone [8]. Mutations in certain genes significantly contribute to the dysfunction of the IKAROS protein, especially in hematological malignancies like lymphoblastic leukemia [9]. Gene deletions, particularly in ALL, have been widely studied [10]. Research on African and Asian populations has explored the relationship between specific *IKZF1* polymorphisms and pediatric ALL. Notably, single nucleotide polymorphisms (SNPs) such as rs4132601 (T > G) and rs11978267 (A > G) have been investigated for their association with ALL in non-western European populations, including live births in England, Wales, Scotland, and the UK [11]. This meta-analysis aims to assess the potential risk of ALL associated with these specific *IKZF1* SNPs, thereby providing insights into their role as biomarkers for the disease.

## Methodology

### Extraction approach and selecting relevant reports for meta-analysis

We systematically searched published literature using databases such as EMBASE, PubMed, Google Scholar, Cochrane Library, and Web of Science, executed in March 2024. The search was performed following the PRISMA guidelines for meta-analysis validation. To identify relevant full-text publications, we limited our search to studies published between 2016 and 2024, employing a combination of keywords and Boolean operators to ensure a comprehensive search. Specifically, the following search terms were used in conjunction with OR/AND Boolean operators: "acute lymphoid leukemia" OR "acute lymphocytic leukemia" OR "ALL," "*IKZF1* gene," "SNP," "association," "polymorphism," "rs4132601," and "rs11978267." The search was confined to titles and abstracts to prioritize studies most likely to meet the inclusion criteria.

To improve the search process's reliability, two independent reviewers carried out both title and abstract screening and full-text screening. Any disagreements between the reviewers were resolved through discussion, and, if necessary, a third reviewer was consulted to ensure consensus. In cases where conflicts arose regarding study inclusion, the reviewers referred to the predefined inclusion and exclusion criteria to guide their decision-making.

We assessed the studies based on predefined inclusion and exclusion criteria. To be included, articles had to meet two key requirements: i) they must have employed a case–control study design, and ii) they must have provided genotype frequency data for both the cases and the controls. Studies were excluded if they met the following criteria: i) lack of control participants, ii) use of animal models, iii) inclusion of cell lines or case reports, or iv) insufficient data. These criteria were applied to select relevant and reliable studies for the meta-analysis. This rigorous screening process ensured that only relevant studies with adequate methodological quality and data were included in the final analysis.

### Data retrieval

Data extraction from the chosen studies included details on study characteristics such as the first author's name, country, publication year, population demographics, sample sizes for both cases and controls, genotypic frequencies of each SNP, and the technology employed for genotyping. Through database searching, a total of 1,148 records were initially identified. After curation based on human genetic studies, 834 records were selected for further evaluation. These records were then screened, resulting in 89 records for detailed review. However, 745 records were excluded due to being focused on non-human genetic studies. Among the remaining records, 44 were related to the *IKZF1* gene polymorphism. Upon further screening, 45 records were excluded due to insufficient data or being reviewed. Ultimately, 13 records were included in the meta-analysis for the SNP rs4132601, as reported in more than three studies, and five records were included for the SNP rs11978267, also reported in over three studies. In total, 18 studies were included in this meta-analysis.

### Assessment of methodological quality using HWE and NOS

The NOS and the HWE were the two criteria the investigators used to assess the quality of the selected analysis. Allocating control genotypes was necessary to comply with HWE (> 0.05). The NOS rating has a maximum possible score of nine points and looks at three factors: revelation, equivalency, and selection. Studies receiving a score of six or higher were considered for inclusion in this meta-analysis.

### Statistical analysis

**Fig. 1:**
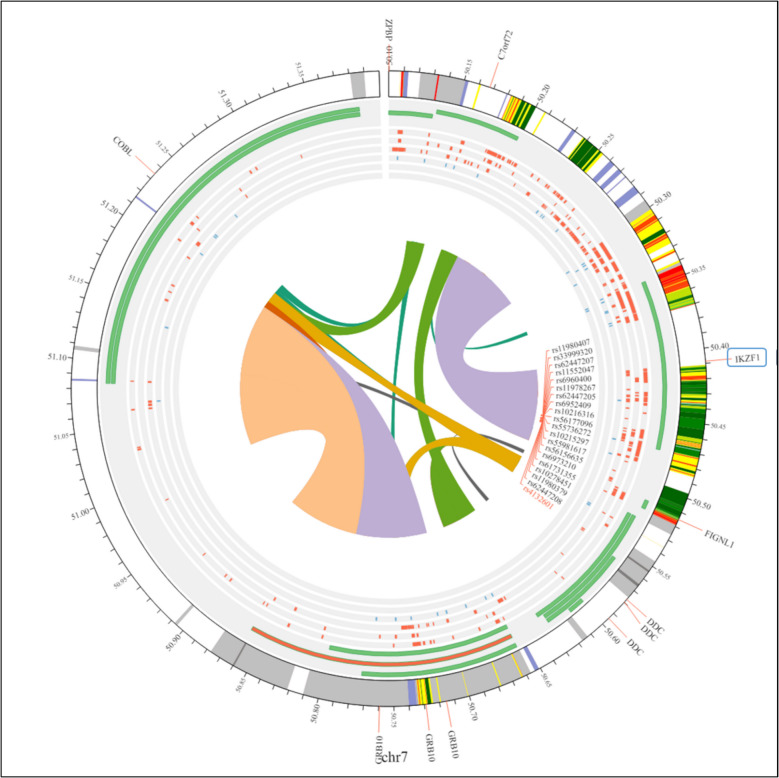
The Circos plot visualizes chromosomal interactions involving the rs4132601 SNP

**Fig. 2:**
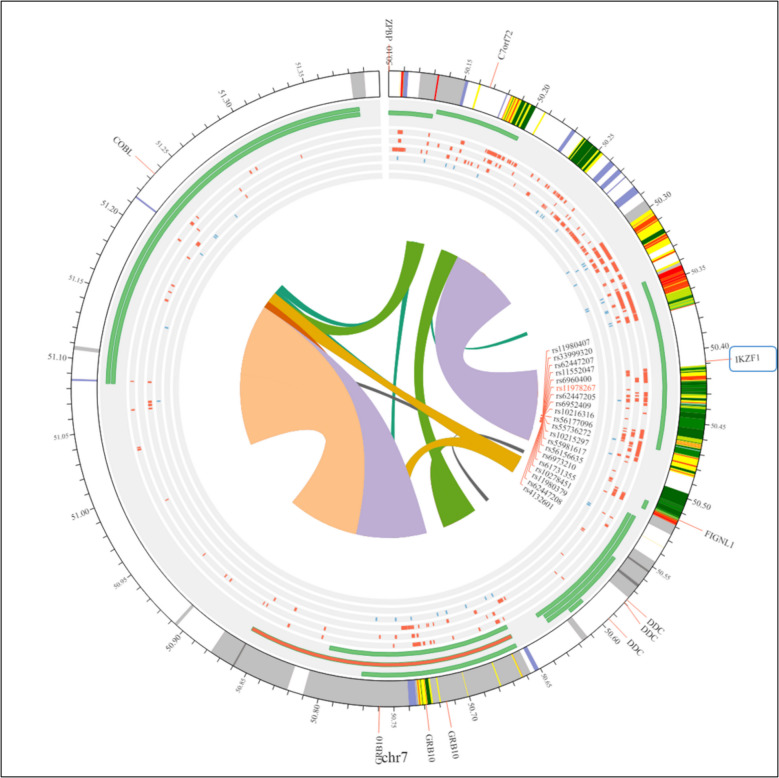
The Circos plot visualizes chromosomal interactions involving the rs11978267 SNP

Data analysis was conducted utilizing Review Manager 5.4 software, and all genetic variations were statistically significant at *p* < 0.05. These tools and methods are essential for carrying out thorough genetic association meta-analyses, evaluating the clinical significance of genetic variants, providing statistical power, and carrying out significance testing in genetic studies on a large scale. The heterogeneity assumption across previous research was interpreted using the Chi-square-based Q statistic test, measured by the *I*^2^ metric value. A significance threshold of *p* < 0.1 was considered noteworthy. The odds ratio and 95% confidence intervals (CI) for previous studies were assessed using the random-effects model. The HWE analysis was carried out using these tools. A funnel plot was utilized to examine publication bias within the meta-analysis. Sensitivity analysis was used to investigate the impact of leaving out certain research, particularly those that followed HWE. The chromosomal interactions with the SNPs are represented by a Circos plot for visualizing the complete data using the 3DSNP tool (https://ngdc.cncb.ac.cn/databasecommons/database/id/1847) in Figs. 1 and 2.

### Protein-to-protein interaction

**Fig. 3:**
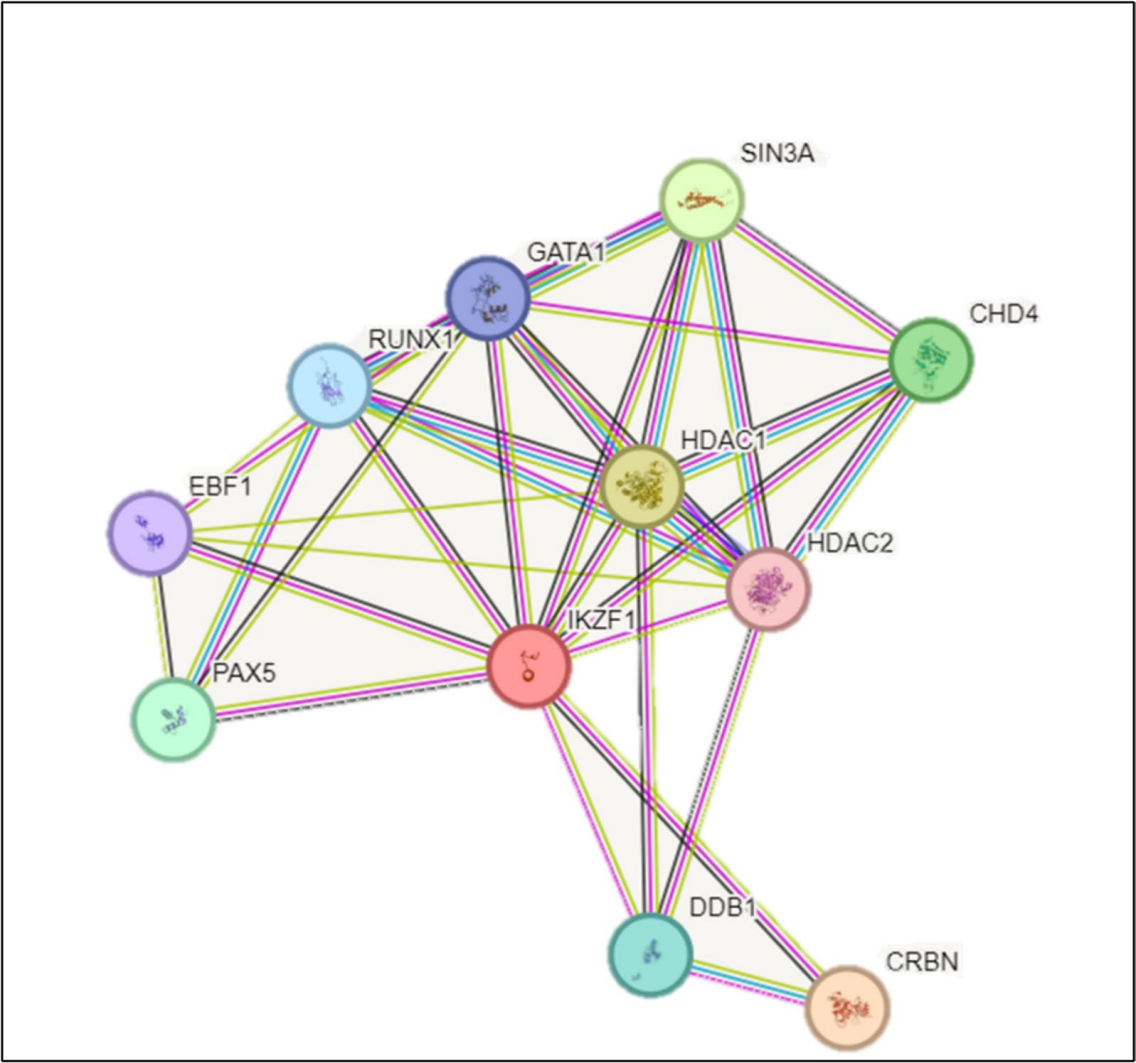
The Protein-Protein Interaction (PPI) network of differentially expressed genes (DEGs) among the selected genes associated with Acute Lymphoid Leukemia

The exploration tool used for searching was the STRING database (Version 11.0; https://string-db.org/), which can forecast functional protein interactions and PPI (protein–protein interaction) scores of ≥ 0.4 associated with ALL and *IKZF1* gene polymorphisms (Fig. 3).

## Results

**Fig. 4:**
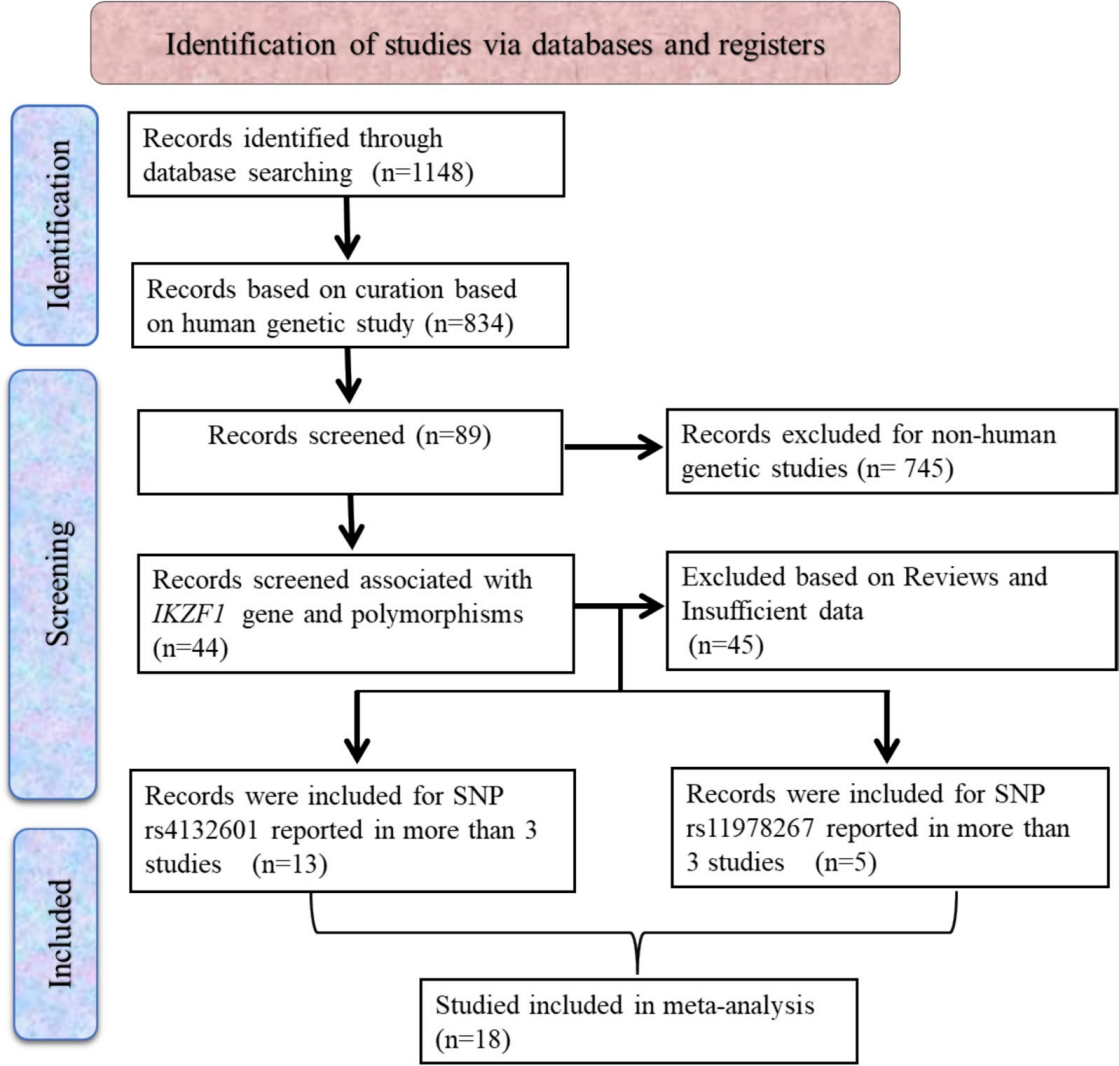
A Flow diagram shows the overview of the study selection

In particular, this study is intended to assess the link between ALL and peculiarities in the *IKZF1* gene. A total of 13 records were included in the meta-analysis for the SNP rs4132601, while five were included for the SNP rs11978267. In total, 18 studies were analyzed in this meta-analysis. We conducted searches in Elsevier's ScienceDirect platform and found thirteen studies with 3546 cases and 6315 controls for the SNP rs4132601 (T > G) [12–24]. Additionally, we found five studies with 952 cases and 1310 controls for the SNP rs11978267 (A > G) [13, 14, 24–26]. Based on the data collected from all thirteen studies, we found an association between ALL and *IKZF1* (rs4132601) gene polymorphisms. In contrast, the data from the five studies showed no association between ALL and *IKZF1* for the SNP rs1197826. The study strategy for examining the *IKZF1* gene is shown in Fig. 4, and information about all the studies we included, including the characteristics of the cases and controls, can be found in Tables 1 and 2. The thirteen studies included diverse ethnic groups.

**Table 1:** Characteristics of study *IKZF1* rs4132601 included in the meta-analysis

Author &Year	Genotypic Frequency						Allele Frequency				Sample size		NOS Scoring	HWE
	**Case**			**Control**			**Case**		**Control**					
	TT	TG	GG	TT	TG	GG	T	G	T	G	Case	Control		
Pastorczak et al. [12]	178	165	46	389	270	56	521	257	1048	382	386	715	7	0.3416
Al-Absi et al. [13]	51	53	32	62	65	26	155	117	189	117	136	153	8	0.2136
Lopes et al. [14]	20	17	2	246	192	29	57	21	684	250	39	467	6	0.2925
Bahari et al. [15]	23	59	28	52	45	23	105	115	149	91	110	120	7	0.0258
Gharbi et al. [16]	49	6	3	98	31	21	104	12	227	73	58	150	7	0.000
Vijayakrishnan et al. [17]	135	52	3	145	36	1	322	58	326	38	190	182	8	0.4355
Sattarzadeh Bardsiri et al. [18]	25	16	9	24	20	6	66	34	68	32	50	50	6	0.5673
Górniak et al. [19]	37	49	14	43	44	13	123	77	130	70	100	100	7	0.7416
Bhandari et al. [20]	69	67	26	78	61	11	205	119	217	83	162	150	8	0.8442
Prasad et al. [21]	531	648	198	1017	707	137	1710	1044	2741	981	1377	1861	6	0.3565
Burmeister et al. [22]	137	151	34	811	574	116	425	219	2196	806	322	1501	8	0.3053
Wang et al. [23]	415	141	12	504	159	9	971	165	1167	177	568	672	7	0.3704
Mosaad et al. [24]	17	23	5	114	66	14	57	33	294	94	45	194	6	0.3067

**Table 2:** Characteristics of study *IKZF1* rs11978267 included in the meta-analysis

Author &Year	Genotypic Frequency						Allele Frequency				Sample size		NOS Scoring	HWE
	**Case**			**Control**			**Case**		**Control**					
	AA	AG	GG	AA	AG	GG	A	G	A	G	Case	Control		
Linabery et al. [25]	224	228	90	204	152	28	676	408	560	208	542	384	7	0.9656
Al-Absi et al. [13]	56	45	35	65	61	27	157	115	191	115	136	153	8	0.06319
Lopes et al. [14]	23	13	3	258	17	34	59	19	533	85	39	309	6	0.0000
Bahari et al. [15]	57	53	0	70	50	0	167	53	190	50	110	120	7	0.0039
Mosaad et al. [24]	32	10	3	122	59	13	74	16	303	121	45	194	6	0.1215
Dong et al. [26]	4	45	141	4	70	196	53	327	74	463	190	270	8	0.4211

**Fig. 5:**
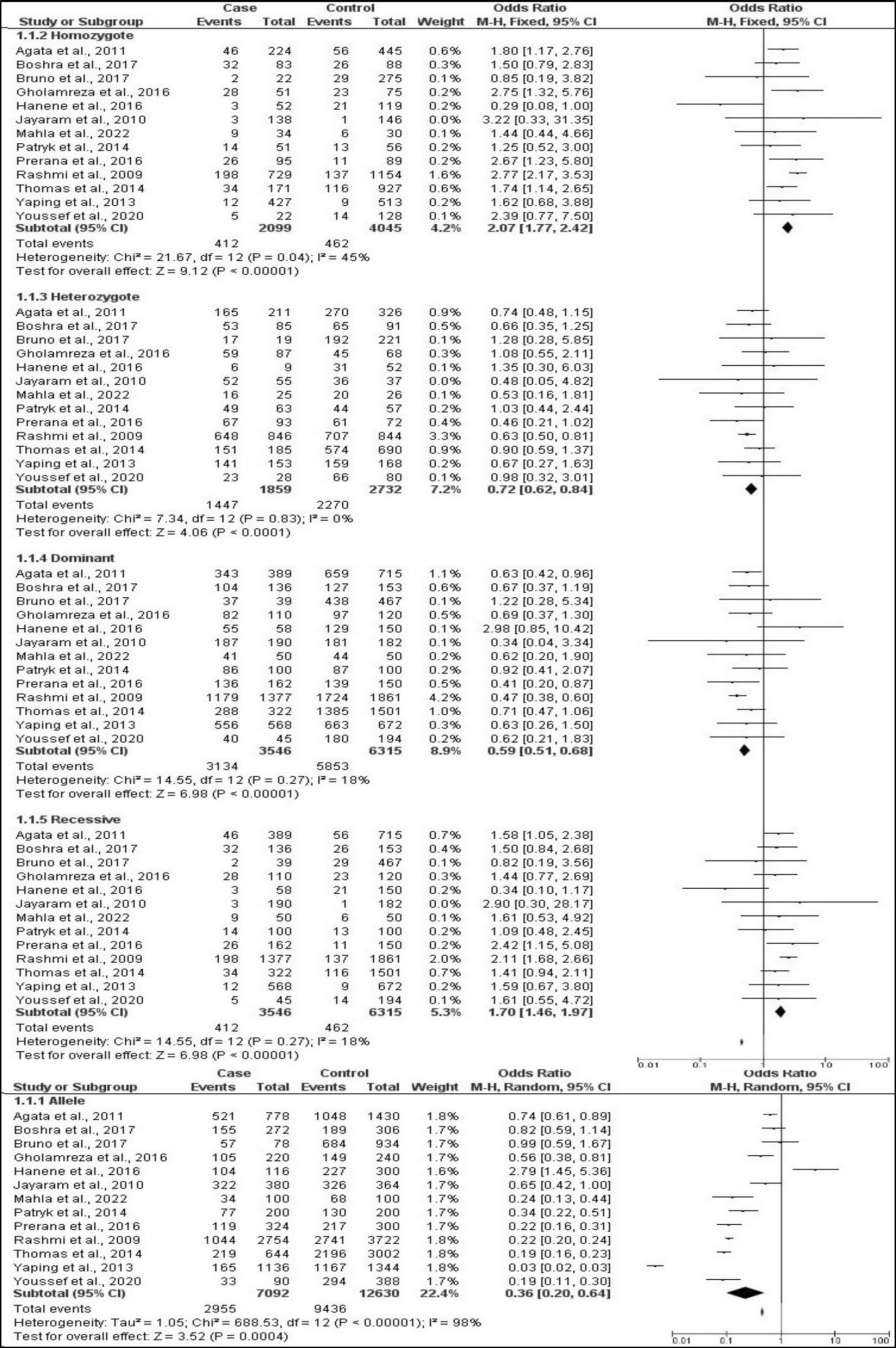
The forest plot shows the heterogeneity in IKZF1 rs4132601 in the fixed and random effects model

It was found that there is a significant link between the *IKZF1* rs4132601 (T > G) gene polymorphism and ALL. The results were obtained using the fixed effects model with an *I*^2^ value of less than 50%. In the homozygous genotype comparison TT vs GG (*I*^2^ = 45%), the OR is 2.07 (95% CI [1.77–2.42]), *p*-value < 0.00001. In the heterozygous genotype comparison TG vs GG (*I*^2^ = 0%), the OR is 0.72 (95% CI of [0.62–0.84]), *p*-value < 0.00001. In the dominant genotype comparison TG + GG vs TT (*I*^2^ = 18%), the OR is 0.59 (95% CI of [0.51–0.68]), *p*-value < 0.00001. In the recessive genotype comparison TT + TG vs GG (*I*^2^ = 18%), the OR is 1.70 (95% CI of [1.46–1.97]), *p*-value < 0.00001. The results were obtained using the random effects model with an *I*^2^ value greater than 50%. In the allelic genotype comparison T vs G (*I*^2^ = 98%), the odds ratio (OR) was 0.36 with a 95% CI of [0.20–0.64] and a *p*-value of 0.0004. These results apply to all study subjects, as shown in Fig. 5.

**Fig. 6:**
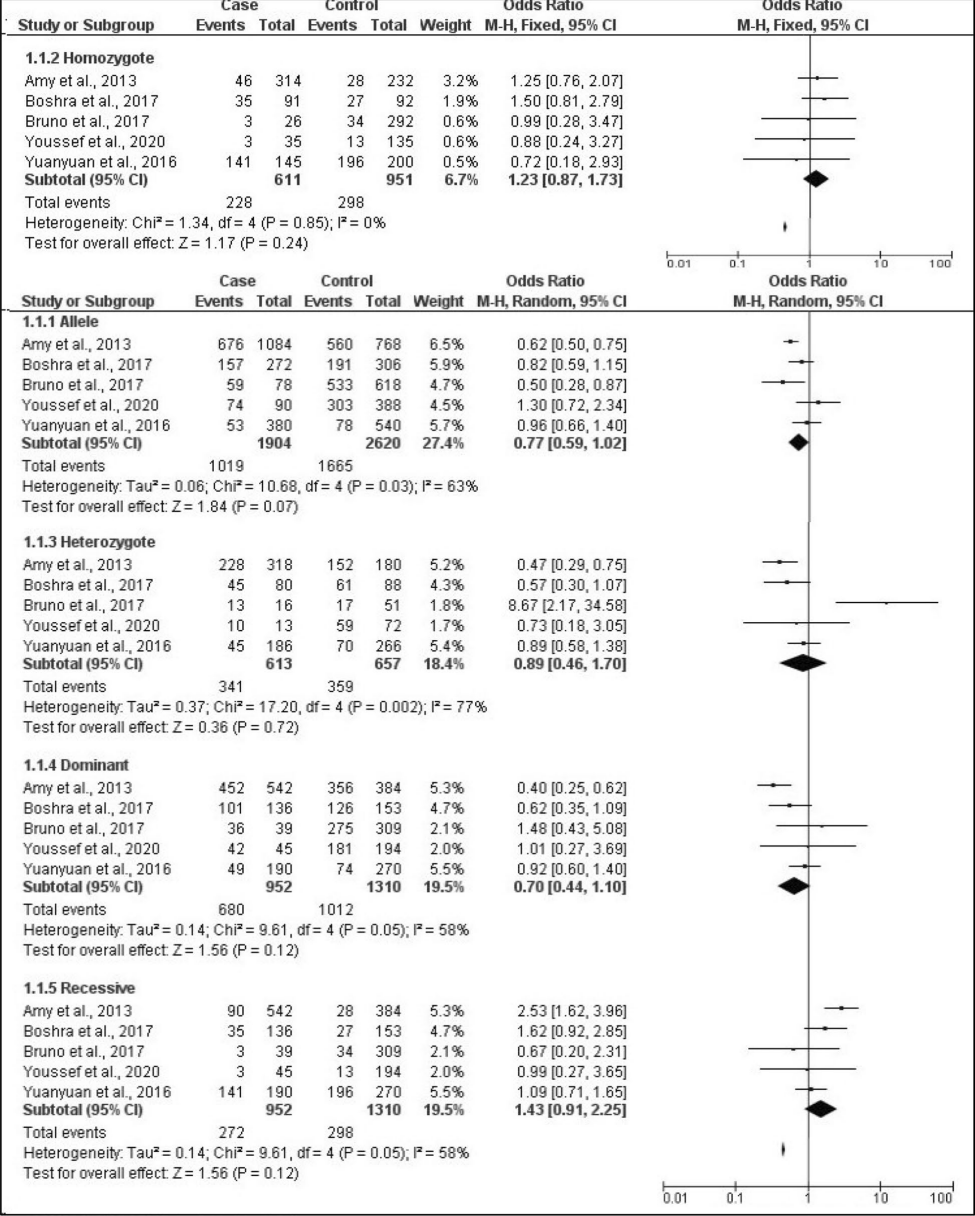
The forest plot shows the heterogeneity in IKZF1 rs11978267 in the fixed and random effects model

**Fig. 7:**
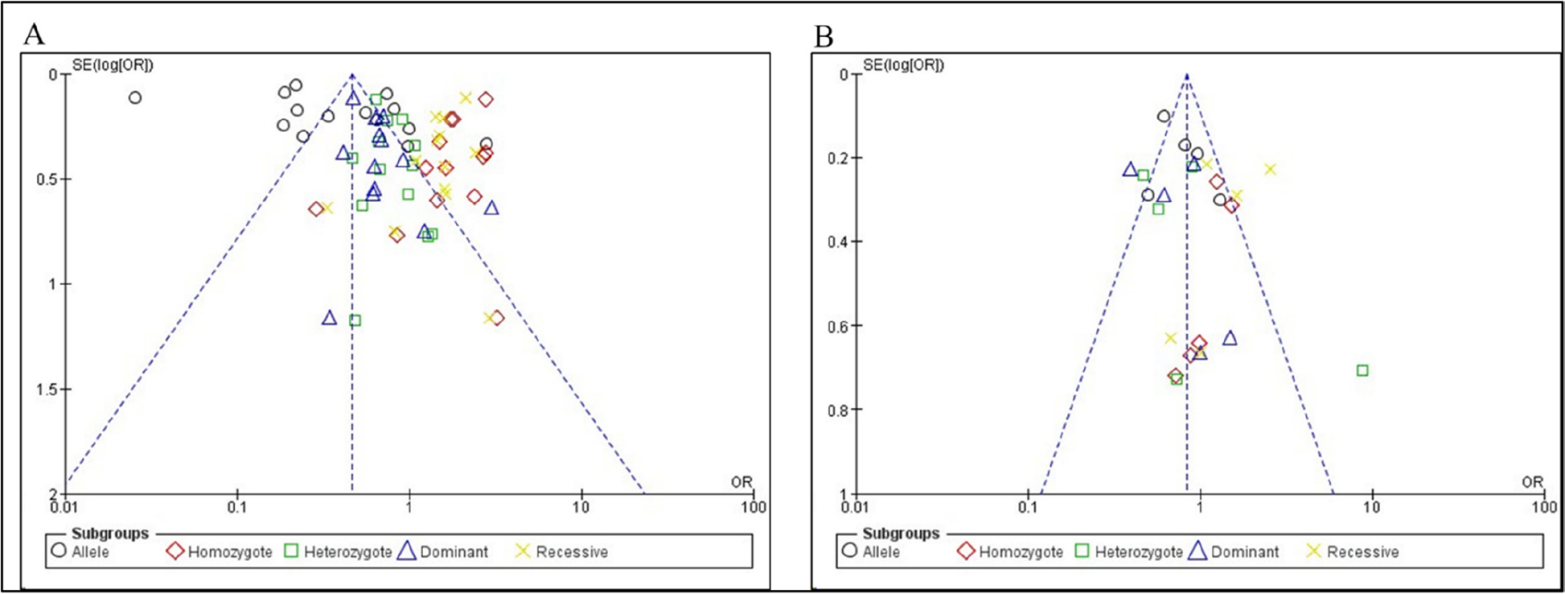
**a** and **b** The funnel plot shows the publication bias for the SNPs ((**a**) rs4132601 and (**b**) rs11978267) in the IKZF1 gene

It was found that there is no substantial link between the *IKZF1* rs11978267 (A > G) gene polymorphism and ALL. The *I*^2^ value was less than 50%, so fixed effects models were used. In the homozygous genotype comparison AA vs GG (*I*^2^ = 0%), the OR was 1.23 with a (95% CI of [0.87–1.73]) and a *p*-value of 0.24. The *I*^2^ value was greater than 50%, so random effects models were used. In the allelic genotype comparison A vs G (*I*^2^ = 63%), the odds ratio (OR) was 0.77 with a (95% CI of [0.59–1.02]) and a *p*-value of 0.07. In the heterozygous genotype comparison AG vs GG (*I*^2^ = 77%), the OR was 0.89 with a (95% CI of [0.46–1.70]) and a *p*-value of 0.72. In the dominant genotype comparison AG + GG vs AA (*I*^2^ = 58%), the OR was 0.70 with a (95% CI of [0.44–1.10]) and a *p*-value of 0.12. In the recessive genotype comparison AA + AG vs GG (*I*^2^ = 58%), the OR was 1.43 with a (95% CI of [0.91–2.25]) and a *p*-value of 0.12. These results apply to all study subjects, as shown in Fig. 6. Begg's and Egger's tests were performed, indicating no evidence of publication bias in this analysis Fig. 7(a) and (b).

A significant connection, with a *p*-value < 0.05, has been found by this meta-analysis between ALL and the *IKZF1* gene rs4132601 across several ethnic groups. For the SNP rs11978267, we located five papers containing 952 cases and 1310 controls. Due to inadequate sample numbers, no link between ALL and the *IKZF1* gene rs11978267 was detected, according to the findings collected in this meta-analysis. Based on the risk bias assessment, the NOS rating considers three factors: revelation, equivalence, and selection, with a maximum achievable score of nine points. For this meta-analysis, studies with a score of six or above were considered.

### An examination of sensitivity analysis

**Fig. 8:**
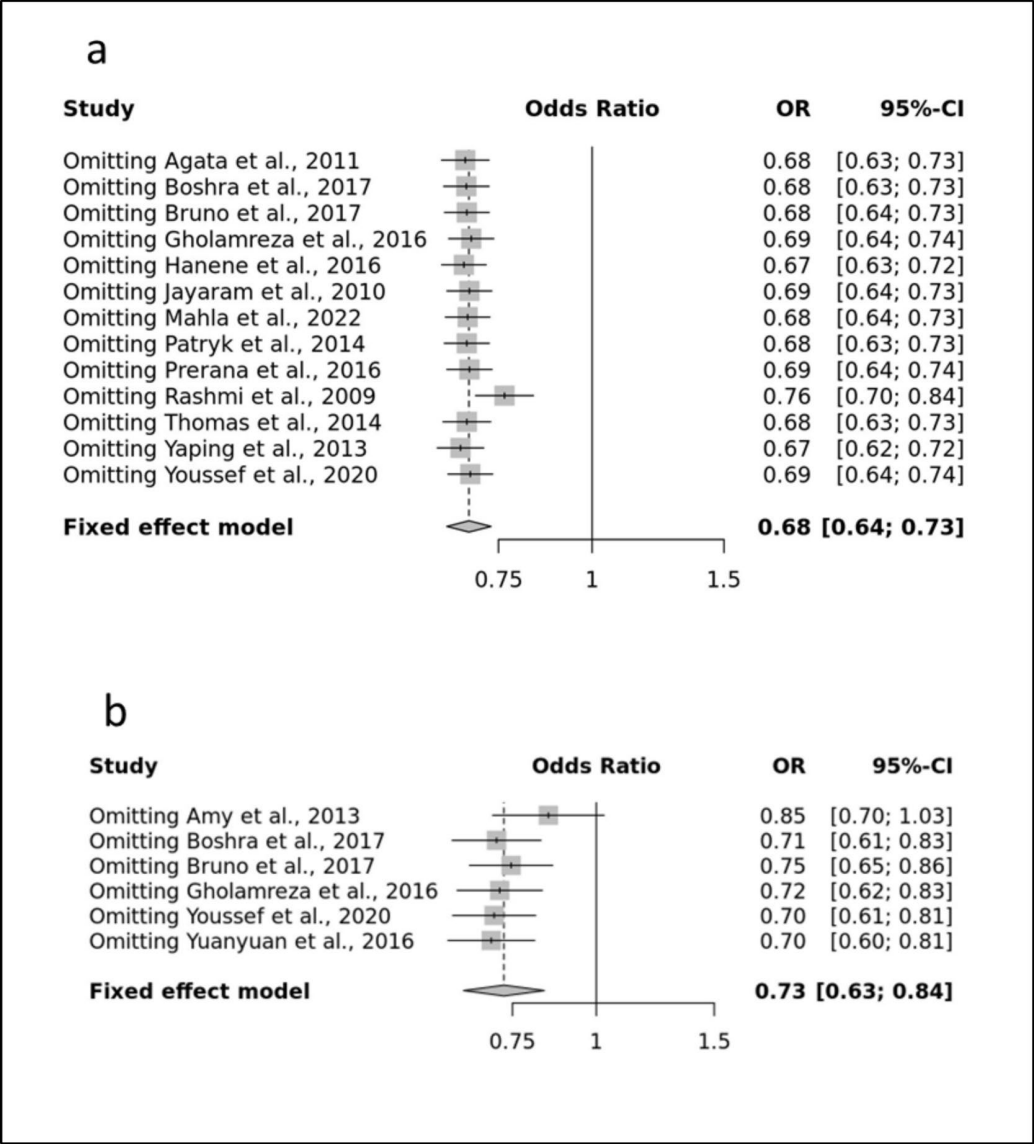
Sensitivity analysis was performed for (**a**) rs4132601 and (**b**) rs11978267 gene polymorphism among ALL cases and controls

We conducted a sensitivity analysis for the genetic variants of the *IKZF1* gene (rs4132601and rs11978267), and Fig. 8(a) and (b) consistently depict unaltered results. This implies that our findings exhibit statistical robustness.

### Evaluation of the PPI network

The PPI network constructed using the STRING database comprises 11 nodes and 34 edges, with an average node degree of 6.18 and a clustering coefficient of 0.817. It exhibits a low PPI enrichment *p*-value of 2e-05, indicating that the proteins interact more frequently than expected in a random set of proteins of similar size and degree distribution from the genome. This enrichment suggests that these proteins are biologically interconnected to some extent as a group. Figure 3 depicts the protein network, highlighting the role of the *IKZF1* gene and its associated proteins in the progression of ALL.

### Evaluation of the Circos plot

The Circos plot was employed to visualize the interactions between genetic loci associated with ALL, focusing on the *IKZF1* gene and its polymorphisms (rs4132601 and rs11978267). This plot offers a detailed overview of chromosomal relationships, gene-environment interactions, and the associations between SNPs across various populations. It illustrates the distribution of *IKZF1* SNPs along chromosome 7, highlighting their proximity to critical regulatory regions of the gene. Both rs4132601 (T > G) and rs11978267 (A > G) are located near these important regions, represented as links in the Circos plot between the SNPs and the corresponding chromosomal segments. The plot also reveals chromosomal regions susceptible to deletions and rearrangements, particularly those impacting *IKZF1*. These deletions have been linked to a poorer prognosis in ALL patients. Furthermore, the plot shows the association of specific SNPs with different clinical subgroups, providing insight into which SNPs are most strongly correlated with the risk of developing ALL in diverse populations (e.g., European, African, Asian). These findings align with previous research identifying *IKZF1* mutations as key prognostic indicators in ALL. Therefore, the Circos plot successfully visualizes the intricate interactions between genetic variations, chromosomal abnormalities, and clinical outcomes in ALL.

## Discussions

ALL is a complex illness that impacts both environmental and hereditary factors. According to GWAS studies, the vulnerability to ALL has been linked to SNPs in 7p12.2 (IKZF1). The SNP at 7p12.2 with rs4132601, situated 3′ to the *IKZF1* gene, showed the highest connection. IKAROS proteins are master regulators that control the commitment of CD4 versus CD8 T-cell lineages during lymphocyte growth and differentiation [27]. The association between ALL and *IKZF1* gene rs4132601 (T > G) showed increased risk in the Iranian population, which was studied by Bahari et al., 2016 [15] and no association was observed with ALL and *IKZF1*gene rs11978267 (A > G) in the same corresponding study [15]. This discrepancy could be due to variations in sample size, study design, or genetic background among different populations. Our meta-analysis addressed these inconsistencies by aggregating data from multiple studies, providing a more robust estimate of the association between the *IKZF1* gene and ALL. The study conducted by Patryk et al., 2014 confirmed the highly significant association between rs4132601 variants located in the 3'UTR region of the *IKZF1* and ALL progression. The major assumption made in the study was that carriers of the GG variant at a younger age of onset could impact the ALL prognosis [19]. The studies were also done to identify the association between ALL and the *IKZF1* gene rs4132601 in Caucasians [17]. In contrast, no association was found among the Asian population [28], which contrasted with the results from other ethnic groups, highlighting the influence of genetic diversity on the disease's progression. These findings underscore the importance of considering population-specific factors when investigating genetic associations in complex diseases like ALL. The study conducted by Sana et al. in 2019 confirmed the association between ALL and *IKZF1* rs4132601, whereas no association was observed among *IKZF1* rs11978267 in the Tunisian population [29]. The study conducted by Youssef et al., 2020 also confirmed the association of ALL and the *IKZF1* gene rs4132601 in the Egyptian case–control population [24].

Evidence of IKAROS's strong tumor suppressor function in pediatric B cell ALL comes from genomic profiling investigations of human leukemia samples, which reveal a high frequency of *IKZF1* mutations and deletions resulting in haploinsufficiency. These mutations may lead to haploinsufficiency, impairing the ability of IKAROS proteins to regulate the differentiation of T and B lymphocytes, thus contributing to leukemia progression. Our study reinforces the idea that genetic alterations in *IKZF1*, particularly rs4132601, may contribute to ALL risk through similar mechanisms across diverse populations [30]. Based on the study observations made by Linabery et al., 2013, an elevated risk of disease to B lineage ALL indicates that rs11978267 is associated with the onset or progression in B cell progenitors during the early phases of differentiation [25].

In this study, we examined a total of 6,315 control individuals and 3,546 ALL patients, using published data to investigate the relationship between *IKZF1* gene polymorphisms and ALL. Our findings reveal a significant association between the *IKZF1* gene polymorphism rs4132601 (T > G) and ALL risk, with a strong allele-wise, over-dominant, recessive, and dominant relationship. This supports the role of rs4132601 as a potential biomarker for ALL susceptibility, which aligns with previous studies conducted in populations from Iran, Egypt, and Caucasians.

However, no significant association was found in studies of Asian populations, including ours, suggesting the influence of population-specific genetic and environmental factors. Additionally, our meta-analysis did not reveal an association between rs11978267 (A > G) and ALL, likely due to the limited sample sizes available in the studies. These results highlight the importance of genetic factors, such as rs4132601, in ALL development but also emphasize the need for more extensive research with larger, more diverse sample sizes to validate these findings. Furthermore, functional studies are crucial to elucidate the mechanisms by which these genetic variants influence ALL pathogenesis. Although our study provides valuable insights into the genetic underpinnings of ALL, further research is necessary to explore gene-environment interactions and their clinical implications for diagnosis and treatment. This meta-analysis indicates a notable correlation between ALL and rs4132601 across various ethnicities (*p*-value < 0.05). However, the association between rs11978267 and ALL remains inconclusive, primarily due to insufficient sample sizes. Larger studies with more diverse ethnic groups are needed to confirm these findings and advance our understanding of the genetic basis of ALL.

## Conclusion

In summary, the relationship between ALL and certain polymorphisms in the *IKZF1* gene SNPs (rs4132601 and rs11978267) was investigated in this thorough meta-analysis. Thirteen studies on rs4132601 and five on rs11978267, covering a wide range of ethnicities, were carefully culled from pertinent data for the study. The rs4132601 gene polymorphism was significantly correlated with ALL, indicating that this variant might contribute to the vulnerability to ALL. Numerous genotype comparisons exhibiting homozygote, heterozygote, dominant, and recessive traits are consistently strongly associated with ALL. On the other hand, as indicated by many genotype comparisons, no meaningful correlation was discovered between ALL and the rs11978267 gene polymorphism. The work highlights the need for additional research with larger sample sizes and varied ethnic communities to corroborate these findings, even though it offers insightful information about the genetic risk of ALL. The meta-analysis emphasizes how complicated ALL is, impacted by genetic variables, and it raises the possibility that future targeted medicines and better diagnostic techniques may result from our understanding of the disease's genetic foundation.

## Data Availability

Not applicable.

## Abbreviations

ALL: Acute Lymphoid Leukemia
BM: Bone marrow
NK cells: Natural killer cells
SNP: Single Nucleotide Polymorphism
*IKZF1* gene: IKAROS family zinc finger 1 gene
NOS: Newcastle Ottawa Scale
HWE: Hardy-Weinberg equilibrium
PRISMA: Preferred reporting items for Systematic reviews and meta-analysis
CI: Confidence intervals
OR: Odds ratio
GWAS: Genome-wide association
